# Complement-targeted therapies in kidney transplantation—insights from preclinical studies

**DOI:** 10.3389/fimmu.2022.984090

**Published:** 2022-10-13

**Authors:** Imran J. Anwar, Isabel DeLaura, Joseph Ladowski, Qimeng Gao, Stuart J. Knechtle, Jean Kwun

**Affiliations:** Duke Transplant Center, Department of Surgery, Duke University School of Medicine, Durham, NC, United States

**Keywords:** complement, allotransplantation, xenotransplantation, animal model, nonhuman primate (NHP)

## Abstract

Aberrant activation of the complement system contributes to solid-organ graft dysfunction and failure. In kidney transplantation, the complement system is implicated in the pathogenesis of antibody- and cell-mediated rejection, ischemia-reperfusion injury, and vascular injury. This has led to the evaluation of select complement inhibitors (e.g., C1 and C5 inhibitors) in clinical trials with mixed results. However, the complement system is highly complex: it is composed of more than 50 fluid-phase and surface-bound elements, including several complement-activated receptors—all potential therapeutic targets in kidney transplantation. Generation of targeted pharmaceuticals and use of gene editing tools have led to an improved understanding of the intricacies of the complement system in allo- and xeno-transplantation. This review summarizes our current knowledge of the role of the complement system as it relates to rejection in kidney transplantation, specifically reviewing evidence gained from pre-clinical models (rodent and nonhuman primate) that may potentially be translated to clinical trials.

## Introduction

### Overview of the complement system

The complement system—made up of many protein mediators, regulators, and cellular receptors **(** **Table 1** **)**—has a diverse range of functions such as immune complex clearance, macrophage activation, opsonization, and modulation of the adaptive immune system (1, 2).

**Table 1:** Major components of the complement system and their function.

	Component	Pathway	Form	Major Functions
**Complement Proteins**	C1q	CP	Soluble	Binds to Fc portion of IgG of IgM in immune complexes. **Initiation of CP**
	C1r	CP	Soluble	Complexes with C1q to cleave C2 and C4
	C1s	CP	Soluble	Complexes with C1q to cleave C2 and C4
	MBL/collectin/ficolins	LP	Soluble	Bind to PAMP. **Initiation of LP**
	MASP-1	LP	Soluble	Dimerizes with MBL/collection/ficolins. Activates MASP-2
	MASP-2	LP	Soluble	Dimerizes with MBL/collection/ficolins. C2 and C4 cleavage
	Factor B	AP	Soluble	Binds to C3b
	Factor D	AP	Soluble	Cleaves Factor B, generating AP C3 convertase (C3bBb)
	C4	CP, LP	Soluble	Cleaved by C1qrs and MASP to C4a and C4b
	C4a	CP, LP	Soluble	Anaphylatoxin
	C4b	CP, LP	Soluble	Complexes with C2a to form C3 convertase (C4bC2a)
	C2	CP, LP	Soluble	Cleaved by C1qrs and MASP to C2a and C2b
	C2a	CP, LP	Soluble	Anaphylatoxin
	C2b	CP, LP	Soluble	Complexes with C4b to form C3 convertase (C4bC2a)
	C3	CP, LP, AP	Soluble	Cleaved by C3 convertase or undergoes spontaneous thioester hydrolysis
	C3 convertase	CP, LP, AP	Soluble	Cleaves C3 to form C3a and C3b
	C3a	CP, LP, AP	Soluble	Anaphylatoxin
	C3b	CP, LP, AP	Soluble	Complexes with C3 convertase to form C5 convertase.
	C5 convertase	CP, LP, AP	Soluble	Cleaves C5 to C5a and C5b
	C5a	CP, LP, AP	Soluble	Anaphylatoxin
	C5b	CP, LP, AP	Soluble	Bind C6, C7, C8, C9 to form MAC
	MAC (C5b-9)	TP	Soluble/Membrane	Lysis, tissue damage, inflammatory response
**Complement Regulatory Proteins**	C1 inhibitor	CRP	Soluble	Irreversible binding to C1r and C1s, inactivation of MASP-1 and MASP-2
	DAF (CD55)	CRP	Membrane-bound	Promotes decay of C3 and C5 converstase. Enhances dissociation of C2 and Factor B
	Protectin (CD59)	CRP	Membrane-bound	Binds to C8 and C9 and prevents MAC formation
	MCP (CD46)	CRP	Membrane-bound	Cleaves C3b and C4b
	Factor H	CRP	Soluble	Downregulates AP *via* C3b inactivation
	Properdin	CRP	Soluble	Upregulates AP *via* C3 convertase stabilization
	Factor I	CRP	Soluble	Inactivates C3b when activated by cofactors
Complement Receptors	CR1 (CD35)	CR	Soluble/Membrane	promotes C3 and C5 convertase decay, C4b and C3b degradation, MASP inhibition, cleaves secretory vesicles
	CR2	CR	Membrane-bound	B cell activation
	CR3	CR	Membrane-bound	C3 split-product-mediated opsonization, T cell regulation
	CR4	CR	Membrane-bound	C3 split-product-mediated opsonization, T cell regulation
	CRIg	CR	Membrane-bound	C3 split-product-mediated opsonization, T cell regulation

There are three pathways through which the complement system is activated: classical, lectin, and alternative. Initiation of these pathways occurs in response to unique molecular signals. The classical pathway is triggered in response to antibody-antigen binding to form an immune complex. C1q is a protein with six globular heads, each of which binds to the Fc region of IgG or IgM, resulting in the complexing of C1q with proteases C1r and C1s. Activated C1s cleaves C4 and C2 into split product C4a, C4b, C2a, and C2b. This allows for the formation of C4b2a, a C3 convertase (i.e. classical C3 convertase) (2). The lectin pathway is initiated following recognition of pathogen-associated molecular patterns (PAMP) and altered self-antigens by mannose-binding lectins (MBL), collectins, and ficolins. The binding activates proteases MASP-1 and MASP-2, in turn cleaving C4 and C2 and resulting in the formation of classical C3 convertase (C4b2a) (3, 4). The alternative pathway is spontaneously activated by constant hydrolysis of the C3 protein. Factor B recognizes C3(H_2_O) and is then cleaved by Factor D to generate C3(H2O)Bb, which in turn cleaves C3 allowing for the generation of C3bBb, the alternative C3 convertase (5, 6). Properdin, a plasma glycoprotein released by neutrophils, monocytes, and endothelial cells in response to stress, stabilizes the alternative C3 convertase. Conversely, factor H, promotes degradation of C3b thus downregulating the alternative pathway (7). All three pathways converge with the creation of a C3 convertase which cleaves C3 into split products C3a and C3b in the common pathway.

C3b complexes with the C4b2a and C3bBb convertases, forming C5 convertases which then cleave C5 into C5a and C5b. C5b complexes with C6-9 to form the terminal membrane attack complex (MAC) (2). The MAC is a cylindrical protein that embeds itself in the cell wall allowing for the entry of extracellular fluid and subsequent cell lysis (8, 9).

Complement split products C3a, C4a, and C5a, also known as anaphylatoxins, serve as mediators of inflammation, resulting in increased vascular permeability, vasodilation, histamine release, and smooth muscle contraction (10, 11). C5a is also implicated in neutrophil chemotaxis. C3b, in addition to its role in the formation of C5 convertase and alternative C3 convertase, binds complement receptors on phagocytes, assisting in opsonization and clearance of immune complexes (11). Anaphylatoxins have their own receptors, C3aR, C5aR1 and C5aR2 (12). C3aR is expressed on neutrophils, basophils, eosinophils, monocyes, mast cells, and certain T and B cell populations, and is involved in chemotaxis of innate cell populations (13–15). C5aR1 is similarly expressed on neutrophils and monocytes, although at higher concentrations than C3aR (16). Additionally, C5aR1 is expressed on T and B lymphocytes and is involved in chemotaxis of both innate and adaptive immune cells (17–20). C5aR2 is involved in modulation of inflammation, and thus innate and adaptive immune responses (21).

Non-anaphylatoxin complement receptors are expressed on a multitude of cell populations and serve a wide range of purposes depending on ligand and cell type, which range from complement system regulation to opsonization to T and B cell modulation. Complement receptor (CR1) is expressed on both innate and adaptive immune cells such as erythrocytes, neutrophils, monocytes, dendritic cells, B and T cells (22). Soluble CR1 (sCR1) arises when membrane-bound CR1 is cleaved from secretory vesicles or the cell membrane (23). CR1 regulates the lectin pathway by binding MBLs and inhibiting MASP activity. CR1 also binds C3b and C4b split products (22). CR2 is a B cell co-receptor expressed on B cells and follicular dendritic cells that binds C3 products and enhances B cell maturation *via* enhanced antigen presentation (24–27). CR3 and CR4 are expressed mainly on monocytes, macrophages, dendritic cells, NK cells, and neutrophils; however, they are also involved in T cell regulation. Primarily, these receptors are involved in C3 split-product-mediated opsonization (28). CRIg is a structurally distinct complement receptor that contains an immunoglobulin domain (29). It is another receptor of C3 split product receptors expressed primarily on tissue macrophages involved in phagocytosis (30).

The majority of complement regulators inhibit proteins at different steps of the complement cascade. C1 inhibitor is a circulating serpin protease inhibitor that primarily prevents spontaneous activation of the classical pathway *via* irreversible binding to C1r and C1s, and the lectin pathway *via* inactivation of MASP-1 and MASP-2 (31). As mentioned above, factor H downregulates the alternative pathway *via* C3b inactivation while properdin upregulates this pathway through C3 convertase stabilization. Factor I is another soluble protein that when activated by certain cofactors facilitates inactivation of C3b (iC3b). Membrane cofactor protein (MCP, CD46; Crry in mice) is one such membrane-bound regulator that disrupts C3 convertase assembly by facilitating factor I-mediated C3b inactivation. Decay accelerating factor (DAF, CD55) and protectin (CD59) are two complement regulators bound to the cell membrane *via* a GPI tail that disrupt the common and terminal pathways respectively. DAF prevents complement activation on self cells by promoting C3 and C5 convertase decay (32, 33). Particularly, DAF enhances dissociation of C2 and factor B, thus blocking all three C3 convertases (34). Protectin binds C9 thus preventing MAC formation (35). Finally, complement receptor 1 (CR1) regulates both the classical and alternate pathway by promoting C3 and C5 convertase decay and cleavage of C3b and C4b (36).

Broadly, complement-targeting therapies act *via* direct downregulation or inhibition of complement elements or potentiation of regulatory proteins thus suppressing various complement functions. As complement activation is implicated in the pathophysiology of many injuries and diseases, such therapies may be applied in a variety of contexts.

### Role of the complement system in rejection in kidney transplantation

**Figure 1:**
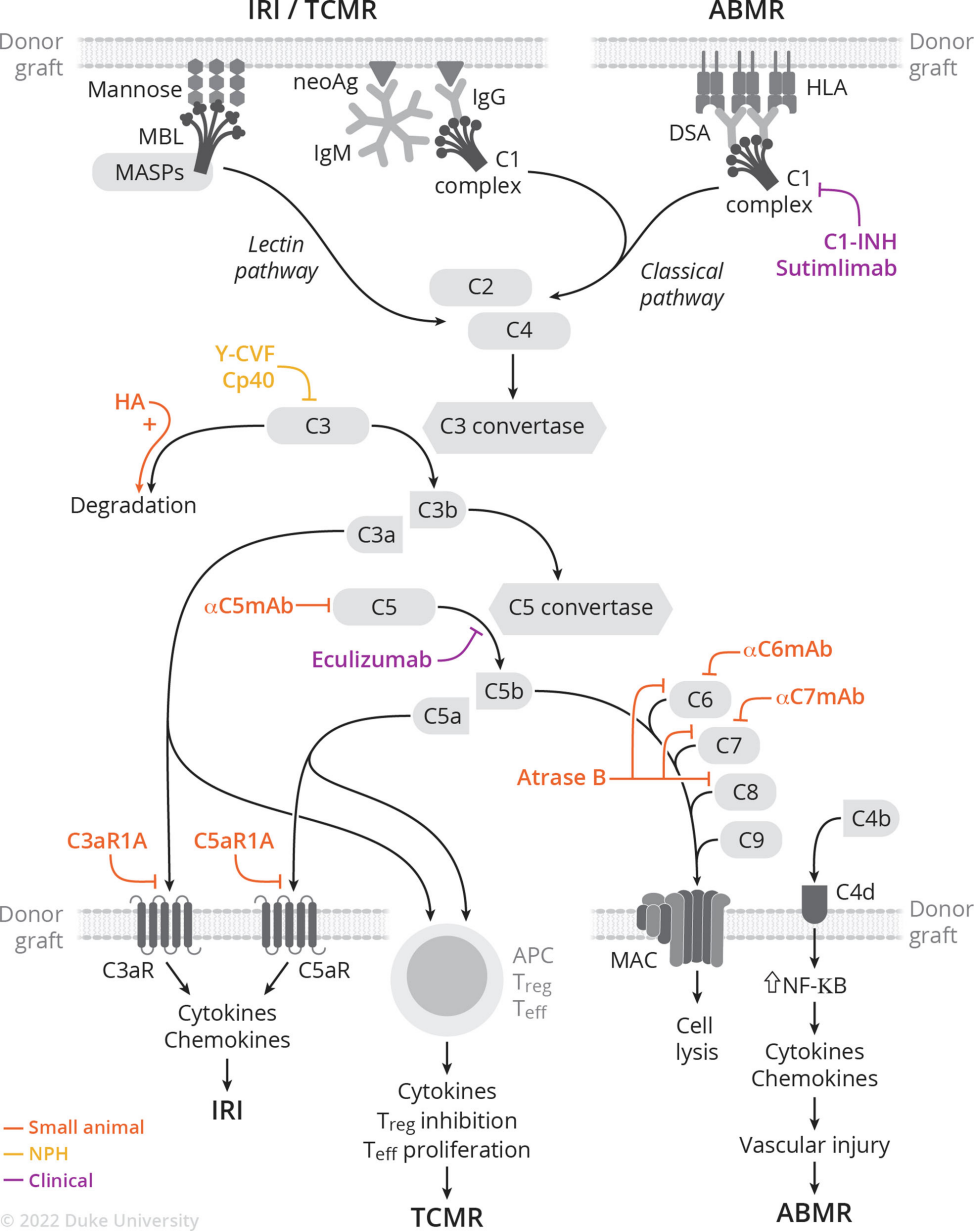
Complement activation cascade and complement-targeting therapies in small animal, NHP, and clinical studies of IRI, TCMR, and ABMR.

The complement system is involved in the pathogenesis of ischemia-reperfusion injury, antibody- and cell-mediated rejection, and vascular injury. Following implantation, ischemia-reperfusion injury generates neo-antigens, which in turn are recognized by either preformed IgM or MBL. This initiates the classical or lectin pathway (**Figure 1**) (37). Additionally, donor-specific antibodies (DSA) bind to donor MHC molecules and initiate the classical pathway (38). This review focuses on the involvement of the complement system in the pathophysiology of rejection; the role of the complement system in ischemia-reperfusion injury (39–42) and vascular injury (43–45) has been recently and extensively reviewed elsewhere.

Under modern immunosuppression, antibody-mediated rejection (ABMR) is the dominant mode of allograft injury (46, 47). DSA bind donor MHC molecules in the allograft, forming immune complexes that activate the classical complement pathway. Following C4 cleavage, C4b covalently binds target sites such as endothelial cells, and is again cleaved into C4d which remains deposited to peritubular capillaries. C4d staining is thus part of the diagnostic criteria for ABMR (46, 48) and is included in routine evaluation of tissue biopsies taken to evaluate for rejection. Importantly, the 2017 Banff criteria incorporates C4d deposition as a surrogate for DSA, highlighting the importance of complement in ABMR pathophysiology and diagnosis (48). In recent years, widespread use of Luminex-based assays has allowed for the precise characterization and quantification of DSA against HLA, in particular allowing for characterization of complement-binding HLA-specific antibodies—those that bind C1q and C3q and activate the complement cascade. Patients with complement-activating DSA are more likely to undergo ABMR, with more extensive microvascular injury and increased C4d deposition on histology (38). These findings were confirmed in a meta-analysis showing that presence of complement-fixing anti-HLA DSA is associated with increased graft loss and allograft rejection, with respective hazard ratios of 3.09 and 3.75 compared to patients with non-complement-fixing anti-HLA DSA (49). Accordingly, the ability to discriminate between complement-fixing (both C1q-binding DSA and C3q binding-DSA) and non-complement-fixing DSA leads to improved prognostication (50, 51). Complement-fixing DSA allows for characterization of one’s response to ABMR treatment, with the presence of C1q-binding DSA portending to increased allograft loss (52). Finally, the complement system is involved in the maturation of B cells in the germinal center, a key step in the humoral response. C3a-C3aR and C5a-C5aR signaling is required for positive B cell positive selection (53).

The complement system is also involved in T cell-mediated rejection (TCMR). Several groups have reported that the complement system is activated during TCMR in kidney transplantation with increased intra-graft C3 mRNA expression (54, 55), as well as increased expression of C1q, C1s mRNA and several complement regulatory genes (55). Furthermore, the complement system is intimately involved with T cell function. It is now well characterized that locally produced C5a and C3a, interacting with their cognate receptors, provide costimulatory signals critical to T cell activation, proliferation, and differentiation (56). Activation of C3a-C3aR and C5a-C5aR promote expansion of the T effector repertoire by suppressing programmed cell death (57). Congruent with those findings, deficiency of DAF accelerates TCMR of cardiac allograft by augmenting strength of the T cell response through pro-proliferative and pro-survival effects on alloreactive CD8^+^ T cells (58). Conversely, genetic or pharmacologic blockade of C3a-C3aR and C5a-C5aR promotes T regulatory cells (59), a well-described subset of T cells involved in limiting immune activation and linked to tolerance (60).

Additionally, the complement system also contributes to graft loss *via* the recruitment of inflammatory cells such as macrophages, neutrophils, and NK cells, which deposit in the graft and can lead to rejection with reduced or absent C4d staining. C5a-CraR1 interactions regulate intragraft migration of suppressive myeloid cells (61). Additionally, sublytic levels of MAC on endothelial cells stimulate NF-κB and subsequent IL-1α and IL-8 production, further increasing inflammation (62–64).

Thus, complement-based therapeutics may be applied to transplantation in the prevention and treatment of multiple pathologies that result in graft dysfunction and failure in kidney transplantation such as ABMR, TCMR, and inflammation. This review focuses specifically on the utility of complement-targeting strategies in kidney transplant rejection.

## Clinical use of complement inhibitors to prevent rejection in kidney transplantation

There are several FDA-approved complement-targeting therapies that have been used off-label in kidney transplantation. C1-INH (Cinryze, Takeda & Berinert, CSL Behring), a recombinant C1 esterase inhibitor, is approved for treatment of hereditary angioedema (65). In February 2022, Sutimlimab, an anti-C1 monoclonal antibody, was approved for treatment of cold agglutinin disease. Finally, Eculizumab (Soliris, Alexion), a humanized monoclonal antibody against C5, is approved for treatment of paroxysmal nocturnal hematuria, atypical hemolytic uremic syndrome, and neuromyelitis optica. These drugs have been used experimentally for ABMR treatment and prevention, as well as to prevent ischemia-reperfusion injury and delayed graft function (66, 67).

### C1 inhibitors

C1-INH has been trialed as ABMR therapy in multiple contexts (68). Vo et al. (69) first reported results of a randomized control trial (RCT) in which C1-INH was administered to highly sensitized patients following desensitization to prevent the development of ABMR. At one month, no patients in the C1-INH group and one in the placebo group had developed ABMR. Following the study endpoint, there was no statistically significant difference in the incidence of ABMR between the two groups, with two patients in the C1-INH and three in the placebo group developing ABMR. Montgomery et al. (70) reported a phase 2b multicenter RCT in which C1-INH was administered as an add-on to standard of care (SOC) for ABMR treatment in 18 patients. There was no difference between groups with respect to pathology or graft survival at day 20 (primary endpoint). However, biopsies taken at six months showed transplant glomerulopathy in three out of seven patients in placebo group, compared to zero out of seven in the C1-INH group, suggesting sequela of chronic ABMR in the placebo group but not in the C1-INH group. Finally, Viglietti et al. (71) performed a prospective, single-arm study to evaluate efficacy and safety of C1-INH in patients with ABMR unresponsive to conventional treatments. Patients underwent six months of treatment with C1-INH and IVIg. All six patients had improved GFR at six months and reduction of complement-fixing DSA. Accordingly, histology showed decreased C4d deposition; however, no other changes in histology were noted. Importantly, a phase 3 multicenter RCT (NCT02547220) to evaluate the efficacy and safety of C1-INH (Cinryze) as an adjunct to SOC for ABMR treatment was terminated after meeting pre-defined criteria for futility. Another phase 3 multicenter RCT (NCT03221842) to evaluate C1-INH (Berinert) as an add-on to SOC for refractory ABMR was discontinued. Therefore, the potential utility of C1-INH as an ABMR therapy is still unclear, given heterogeneous study outcomes, differing drug regimens, and small sample sizes (68).

Sutimlimab (Enjaymo, Sanofi), an anti-C1 mAb, was recently evaluated in a phase 1 clinical trial (72). Ten kidney transplant recipients with evidence of late active ABMR and classical pathway activation (C4d deposition, complement-fixing DSA) received four weekly doses of sutimlimab. All 10 patients had improvement in C4d deposition: five out of eight turned C4d-negative while the remaining two patients had a decrease in their C4d scores. There were overall no changes in renal function or DSA.

### C5 inhibitors

The use of C5 inhibitors in kidney transplantation was first described in 2009 by Dr. Montgomery’s group, who reported a case of eculizumab and splenectomy as salvage therapy for severe ABMR following HLA-incompatible transplant (73). Orandi et al. (74) expanded on the use of eculizumab and splenectomy as salvage therapy: five out of five patients treated with eculizumab/splenectomy/plasmapheresis saw graft survival and minimal transplant glomerulopathy at one year. However, four out of five patients treated with eculizumab/plasmapheresis had graft failure and TG on histology at one year. Stegall et al. (75) explored the utility of eculizumab as ABMR prevention in HLA-incompatible kidney transplantation. Twenty-six highly sensitized recipients received eculizumab post-transplant, and ABMR rates were compared to historic cohorts. ABMR incidence (7.7% *vs*. 41.2%) and rates of TG on one-year biopsy (6.7% *vs*. 35.7%) were lower in the eculizumab group compared to historical cohort. However, long-term follow-up of this cohort revealed no differences on one-year protocol biopsies, suggesting that eculizumab does not prevent chronic ABMR in patients undergoing HLA-incompatible transplants (76). A phase 2 multicenter RCT (NCT01399593) to assess safety and efficacy of eculizumab as ABMR prevention following HLA-incompatible kidney transplantation was terminated due to lack of superiority at primary endpoint (biopsy‐proven acute ABMR, graft loss, death, or loss to follow‐up) (9.8% in eculizumab group *vs*. 13.7% in SOC, p=0.76) (77). However, biopsies were evaluated by a central pathologist without clinical information at hand; *post-hoc* reassessment by central pathologists with clinical context yielded a larger observed difference between eculizumab and SOC (11.8% and 21.6%, p=0.28) and significant difference if grade 1 AMR scores were included (11.8% *vs*. 29.4%, p=0.048). Interestingly, another phase 2 (NCT01567085) open label single-arm multicenter trial with 80 highly sensitized patients undergoing deceased-donor kidney transplantation and post-transplant receiving a 9-week course of eculizumab showed a 8.8% treatment failure rate at 9 weeks post-transplant, compared to 40% expected treatment failure rates with SOC (78). In this study, only 6.3% of eculizumab treated patients showed histological evidence of ABMR within 12 months (78). Unfortunately, this study’s conclusion is dampened by the single-arm design and the use of central pathology biopsy assessment without clinical information or grade 1 ABMR for primary end point decision. In summary, the evidence in support of eculizumab as ABMR salvage or prevention remains unconvincing.

Overall, clinical trials of complement-based therapies have yielded mixed outcomes, and interpretation of these trials has been limited by small sample sizes, heterogeneity in treatment regimens, and varying definitions of ABMR. Nevertheless, several promising complement-based therapies have been investigated in pre-clinical models that may yield translatable strategies in humans. The rest of this review summarizes promising complement-based strategies tested in pre-clinical models that offer potential solutions for organ rejection in humans.

## Rodent studies

### Common pathway

The three initial complement pathways all converge on the formation of a C3 convertase. C3 split products play multiple important roles, such as C5 convertase formation, opsonization, inflammatory mediation, and alternative pathway activation; thus, C3 is an appealing target for inhibition with the potential to quiet the entire complement cascade and several of its functions. In 2002, Pratt et al.’s breakthrough paper elucidated the role of C3 in modulating acute rejection and regulating T cell responses in a C3 knockout (KO) murine kidney transplantation model, further supporting the potential of C3 as a therapeutic target (79). The transplanted C3 KO mice had longer graft survival compared to the WT controls, with less tubular and vascular inflammation on graft histology. Furthermore, C3 KO mice displayed defective T cell priming, and *in vitro* experiments showed the activation of T cell complement receptors CR1 and CR2 by fragments C3b, iC3b, C3dg, and C3d (80). This paper put forth several hypotheses regarding the role of C3 in T cell activation. First, C3 may bind covalently to tissue, thus activating alloreactive T cells through complement receptors—a response not seen in C3 KO tissues. Second, antigen processing and display by APCs may be more efficient in response to C3-opsonized antigens. Lastly, complement-activated APCs may have higher expression of MHC and costimulatory molecules, resulting in more robust T cell activation.

C3 has also been targeted in small animal xenotransplantation: Malassagne et al. assessed the impact of C3 degradation on hyperacute rejection in a guinea pig to rat heterotopic heart transplant model (81). Hypodermin A (HA), a serine esterase, was administered prior to transplant to trigger C3 degradation and was found to significantly delay onset of hyperacute rejection. *In vitro* experiments showed lower deposition of terminal complement products C6 and MAC in HA-transfected cells, while serum CH50 levels decreased with HA administration *in vivo*.

Several rodent studies have used blockade of other steps in the common complement pathway in transplantation. For example, anti-C5 mAb treatment in a murine heart transplant model resulted in increased graft survival (82). C5 blockade inhibits the proinflammatory actions of anaphylatoxin C5a and MAC, C5b-9, without inhibiting upstream proteins. The combination of anti-C5 mAb and cyclosporine resulted in reduction of DSA below detection threshold and indefinite graft survival, with less evidence of acute vascular rejection and acute cellular rejection on histology. Interestingly, long-term survivors had low levels of antibody deposited in the allograft. Anti-C5 mAb treatment in a pre-sensitized heterotopic heart transplantation model resulted in permanent graft survival with no evidence of rejection on histology, despite persistently elevated DSA titers and complement levels (83). Interestingly, the authors rechallenged recipients with re-transplantation and found that accommodation required C5-depletion of both the graft and recipients.

Furthermore, C5 blockade, in conjunction with costimulation blockade, prevents TCMR in a heterotopic heart transplant murine model by limiting induction of donor-specific T cells and inhibiting the response of allo-primed T cells (84). C5 blockade also reduced trafficking of allo-primed T cells to the graft.

Both activation of the classical and lectin pathways leads to cleavage of C4 into C4a and C4b. As such, C4 constitutes an appealing target to prevent activation of the complement system. Li et al. (85) surprisingly found no effect of C4 knockout, either in the recipient or donor, on allograft survival and alloantibody responses in a murine model of MHC mismatched kidney allotransplantation. Presumably, complement activation seen in their model resulted from activation of the alternative pathways and underlines the importance of blocking all 3 pathways to achieve meaningful blockade of the complement system.

Therefore, targeting elements that are common to all 3 complements pathways—namely *via* inhibition of C3 and C5—has resulted in robust and effective suppression of multiple immune processes in small animal models, such as prevention of hyperacute rejection, TCMR, ABMR, and graft infiltration.

### Terminal pathway

The membrane attack complex (MAC), also known as terminal complement complex (TCC), constitutes the end-product of the complement cascade. MAC deposition on the cell surface of non-nucleated cells disrupts the cell membrane, allowing for influx of fluid, and subsequent cell lysis and death. In nucleated cells, MAC leads to release of pro-inflammatory cytokines and enhanced the immunogenicity (86).

Deficiency of C6, one of the proteins that forms the MAC, results in prolonged graft survival in a model of long-term cardiac allotransplantation model (87). Heterotopic heart transplants were performed between MHC-mismatched rats with either WT or C6 deficient donors. Cardiac allografts in the C6-sufficient recipient rejected between days 21-84 with evidence of vascular lesions from endothelielitis to obliterative arteriopathy in the longest survival grafts, in contrast with rejection between days 60-120s in the C6-deficient recipients and minimal vascular lesions.

MAC-targeting therapies have been extensively used in xeno-transplant studies in small rodents. Using a hamster-to-rat heart heterotopic xenotransplantation model, Suhr et al. (88) demonstrated that genetic or antibody-induced C6 deficiency leads to accommodation, highlighting the potential therapeutic potential of downstream complement targeting. Another terminal pathway inhibitor, Atrase B, is a metalloproteinase isolated from the venom of Naja Atra that potently cleaves complement components C6, C7, and C8, effectively suppressing the terminal pathway while leaving the rest of the complement system intact. Atrase B delays xenograft rejection in a Guinea pig-to-rat heterotopic heart transplantation model (89). Importantly, the authors noted anti-coagulant properties of Atrase B as evidenced by prolonged PT and aPTT, as well as decreased platelet microthrombi and fibrin deposition in the graft. This anticoagulant effect offers a potential benefit given the marked coagulation issues (i.e., thrombotic microangiopathy) described in pig-to-NHP xenotransplantation.

Despite these promising studies, MAC-targeting therapies have yet to be utilized in humans or in non-human primates. This may be in part due to the reliance of experiments on genetic modifications or compounds that are not readily translatable to humans (e.g., venom). Several groups recently described novel monoclonal antibodies that target the MAC: Lin et al. (90) developed a monoclonal antibody that inhibits MAC formation by blocking both free C6 and C6 in the C5b6 complex. Zelek et al. reported the generation of several novel blocking C7 monoclonal antibodies effective across multiple species, including humans. These new antibodies may lay the groundwork for potential translation of MAC-based therapies.

### Anaphylatoxins and their receptors

Complement split products are released following activation of the complement cascade. The cleavage of C3 and C5 by their respective convertases results in split products C3a and C5a, which play a major role in inflammation and activation of immune and non-immune cells. C3a and C5a receptors (C3aR and C5aR, respectively) are G protein-coupled receptors that are expressed both in myeloid and non-myeloid cells, where they participate in regulation of the adaptive immune response (91). As such, interventions that target C3a-C3aR and C5a-C5aR may be beneficial in the setting of transplantation.

When combined with tacrolimus, absence or blockade of recipient C3aR1 prolongs cardiac allograft survival in an MHC-mismatched model of heterotopic heart transplantation. This effect was at least partially mediated by reduced expansion of donor-reactive CD8^+^ T cells, thus more broadly inhibiting the donor-reactive T cell repertoire (92).

Deficiency of C5aR, in either the recipient or donor, prolonged renal allograft survival in a fully MHC-mismatched mouse model (93). Allo-specific T cell proliferation and cytokine production, as well as antigen presenting cell (APC) function (of both donor and recipient) were impaired in the C5aR deficient group. Furthermore, absence of C5aR in both the donor and recipient led to decreased cellular infiltration of the graft. Similar findings were reported with the use of a C5aR antagonist in an MHC-mismatched mouse kidney transplantation model (94). Pre-transplant treatment with a C5aR antagonist led to long-term survival while immediate post-transplant treatment delayed but did not prevent graft failure. The salutary effects of C5aR antagonism are mediated by reduced cellular infiltration of the graft, as well as robust decrease of alloreactive T cell priming. Importantly, a first-in-class C5aR inhibitor, Avacopan (Tavenos, Chemocentryx), was approved by the FDA in October 2021 for ANCA-associated vasculitis, paving the way for potential use in transplantation.

## Nonhuman primate studies

### NHP allotransplantation

Several studies investigated the use of complement-based therapies in nonhuman primate (NHP) models of allo- and xeno-transplantation. A brief course of compstatin (Cp40), a C3 inhibitor, prolonged graft survival and prevented antibody-mediated graft injury in a highly sensitized NHP allotransplantation model. (95). This study did not utilize antibody-reducing desensitization strategies but nevertheless saw prevention of early ABMR with rhesus ATG induction, triple immunosuppression, and the addition of C3 inhibition. Notably, normal graft function was maintained despite the presence of elevated DSA, further supporting the importance of complement blockade in improving transplantation outcomes and the potential to induce accommodation. Despite significantly prolonging graft survival, all animals ultimately developed graft failure in the setting of ABMR. Animals that developed early graft failure had higher levels of IgM, suggesting that the IgM-antigen interaction likely promoted breakthrough complement activation under Cp40 therapy. Further studies are thus needed to assess whether a higher Cp40 dosing regimen or longer course would durably prevent ABMR. C3 was also targeted in a 2011 study, which reported successful prevention of ABMR and subsequent accommodation and long-term survival in sensitized NHPs treated with Yunnan cobra venom factor (Y-CVF) (96). Y-CVF treatment results in depletion of circulating C3 and robust complement blockade (97). Chen et al. showed DSA suppression and survival past 1,000 days in multiple sensitized NHPs treated with Y-CVF and triple immunosuppression. These studies indicate the potential utility of C3 blockade in reducing ABMR and inducing accommodation.

C1-INH has been used to prevent ABMR in NHP studies as well: Tillou et al. (98) sensitized baboons with two PBMC injections from allogeneic donors prior to kidney transplant from the same donor. Rapid acute ABMR ensued following transplantation and untreated animals rejected within 48-72 hours post-transplant. C1-INH was given during the first five post-transplant days in the experimental group, which successfully prevented rejection during the treatment period. However, rejection occurred following C1-INH discontinuation. In a NHP model of kidney allotransplantation after brain death and prolonged cold ischemia, animals treated with recombinant human C1-INH displayed less delayed graft function and also trended towards prolonged antibody-mediated free graft survival (99).

### Genetic modifications in xenotransplantation

Xenotransplantation, the use of pigs as organ donors, provides a unique opportunity to genetically engineer the donor organ. Early xenotransplantation studies were limited by hyperacute antibody-mediated rejection (HAR) and rapid graft failure; genetic modification of the donor pig has proved necessary for meaningful graft survival. Antigen deletion and gene insertion—specifically expression of human complement-regulatory proteins (CRP) on the pig endothelium—are two strategies that have been utilized to prevent rejection. These two strategies are not mutually exclusive and many of the pigs used in the pre-clinical trials of pig-to-non-human primate xenotransplantation possess a combination of gene deletion and CRP expression modifications.

Antigen deletion is arguably the preferred approach to xenotransplantat modifications, as the lack of antigen prevents the initiation of the complement cascade, and CRPs are theoretical pathogen co-receptors (100). In total, three carbohydrate xenoantigens have been identified in the pig genome (αGal, Neu5Gc, and the SDa antigen) (101–103). The first xenoantigen, αGal, has been knocked out in pigs *via* a mutation in GGTA1, the enzyme responsible for αGal, thus extending graft survival significantly in early pig-to-non-human primate (NHP) trials (104, 105). Knockout of the second xenoantigen, Neu5Gc, further decreases human antibody against pig cells; however, the impact of this modification is difficult to evaluate in a NHP model as Old World primates possess the responsible enzyme, CMAH, and a CMAH-KO pig worsens the pig:NHP crossmatch (106, 107). The enzyme responsible for biosynthesis of the third recognized xenoantigen, the SDa antigen, is β4GalNT2 (108). Human antibody against cells from pigs with mutations in the responsible enzymes [GGTA1, CMAH, and β4GalNT2 respectively, termed “triple-knockout” (TKO) pigs] approaches background levels in a proportion of the population (109). Other proposed xenoantigen targets are swine leukocyte antigen class I and II, though there has been no survival benefit to a class I KO pig in the limited trial reported (110–112).

In the early phase of xenotransplantation genetic engineering, CRP expression was essential to prolonging graft survival. The three primary CRPs studied in xenotransplantation target different portions of the complement cascade: membrane cofactor protein (CD46) cleaves C3b and C4b with serum factor I, decay accelerating factor (CD55) prevents formation of the C3 convertase (C4b2a), and protectin (CD59) inhibits C9 and membrane attack complex (MAC) formation. Given the difficulty of genetic engineering, breeding, and raising large animals, it has been difficult to directly compare outcomes with CRP alterations, but some conclusions can be drawn. The earliest success with CRP modifications came with CD55-transgenic pigs: NHP trials demonstrated kidney graft survival comparable to GGTA1 single knockout animals (113–115). Next, CD59-transgenic pigs were developed, but ultimately failed to significantly increase survival (116). Finally, CD46-transgenic pigs were produced, (117) and, like CD55 pigs, also extended graft survival. As a result, CD55 and CD46 knock-ins have been further utilized in preclinical studies.

It became widely recognized, particularly in cardiac xenotransplantation, that the combination of CRP(s) and antigen deletion was necessary to prevent early graft losses (118, 119). To this point, the single long-surviving cardiac xenograft in pre-clinical literature, at over 900 days, was a multi-gene transgenic pig (120), and the recent first-ever pig-to-human life-supporting cardiac xenotransplantation was also from a transgenic pig. The mechanism behind the benefit of CRP modifications is unclear, as kidney xenotransplantation studies have demonstrated some success without CRP alterations (121), though this may be an organ-specific phenomenon.

### Pharmacologic interventions in NHP xenotransplantation

In addition to genetic modifications aimed at minimizing discordance between the human and xenograft complement system, complement-targeting regimens are commonly used to prevent rejection episodes.

C1-INH successfully reversed acute vascular xenograft rejection (AVR) in a pig (h-DAF knock-in)-to-cynomolgus life-supporting kidney xenotransplantation model. C1-INH was added to cyclophosphamide/steroids and successfully reversed six out seven AVR episodes. In contrast, cyclophosphamide/steroids alone failed to reverse any AVR episodes (122).

Y-CVF was also trialed in pig (wild type)-to-rhesus heterotopic heart transplant model. Addition of Y-CVF to cyclosporine A, cyclophosphamide, and steroids successfully prevented hyperacute rejection in four out of four animals. However, continuous treatment did not ultimately prevent AVR, with all grafts rejecting prior to two weeks post-transplantation (97).

Finally, Adams et al. (112) expanded on the utility of anti-C5 to prevent early ABMR in pig-to-rhesus kidney xenotransplant. Donor kidneys were harvested from either double-knockout (Gal, Sd_a_) or triple-knockout (Gal, Sd_a_, SLA I) pigs. Temporary therapy (up to 70d) of anti-C5 mAb improved graft survival potentially due to reducing rates of early ABMR.

## Future directions and conclusion

The pathological involvement of the complement system in kidney transplantation has gained importance in the last decades and it is now well established that complement activation leads to rejection both in allo- and xeno-transplantation. Efforts to dampen the complement-mediated response have thus far enjoyed mixed results in clinical trials. However, preclinical studies hint at promising new therapeutic approaches that could be readily translated to humans. C3 blockade remains the most promising therapeutic avenue given its central role in the amplification of the complement cascade. Furthermore, C3 blockade has been tested in NHP and successfully prevents acute ABMR in a stringent sensitized model (95). Additionally, the FDA recently approved Pegcetacoplan, a C3 inhibitor, for paroxysmal nocturnal hematuria and phase II trials are currently ongoing regarding use in kidney transplant recipients with recurrent C3 glomerulopathy (NCT04572854). MAC-based therapies have thus far not been evaluated in NHP studies or clinical trials despite promising rodent studies both in allo- and xeno-transplantation. Several new monoclonal antibodies targeting components of the MAC have recently been generated (90, 123) and could pave the way for future studies. Finally, blockade of the C5a-C5aR axis successfully prevents rejection in rodent studies by preventing allo-specific T cell proliferation and priming, APC function, and graft infiltration. Avacopan, a C5aR inhibitor, is FDA-approved for ANCA-associated vasculitis and could thus be readily used in kidney transplantation. Overall, complement blockade offers a promising approach to ABMR prevention and treatment, requiring further preclinical and translational study.

## Author contributions

All authors contributed to the article and approved the submitted version.

## Funding

This work was partially supported by the National Institute of Allergy and Infectious Diseases of the National Institutes of Health as part of the NHP Transplantation Tolerance Cooperative Study Group under the U19AI131471 (awarded to SK) and Opportunities Pool Round 16 (awarded to JK). Additionally, this work was supported by NIH T32 AI141342-03 (awarded to IA) and ASTS Presidential Student Mentorship Grant (awarded to ID).

## Conflict of interest

SK has been a consultant to Novartis, Sanofi, and Viela Bio. SK and JK received research grants from Alexion, MorphoSys, and eGenesis.

The remaining authors declare that the research was conducted in the absence of any commercial or financial relationships that could be construed as a potential conflict of interest.

## Publisher’s note

All claims expressed in this article are solely those of the authors and do not necessarily represent those of their affiliated organizations, or those of the publisher, the editors and the reviewers. Any product that may be evaluated in this article, or claim that may be made by its manufacturer, is not guaranteed or endorsed by the publisher.

## References

[1] Schifferli JA Ng YC Peters DK. The role of complement and its receptor in the elimination of immune complexes. N Engl J Med (1986) 315:488–95. doi: 10.1056/NEJM198608213150805 2942776

[2] Walport MJ. Complement. First of two parts. N Engl J Med (2001) 344:1058–66. doi: 10.1056/NEJM200104053441406 11287977

[3] Wallis R. Interactions between mannose-binding lectin and MASPs during complement activation by the lectin pathway. Immunobiology (2007) 212:289–99. doi: 10.1016/j.imbio.2006.11.004 PMC2592599 17544814

[4] Garred P Genster N Pilely K Bayarri-Olmos R Rosbjerg A Ma YJ. A journey through the lectin pathway of complement-MBL and beyond. Immunol Rev (2016) 274:74–97. doi: 10.1111/imr.12468 27782323

[5] Lachmann PJ. The amplification loop of the complement pathways. Adv Immunol (2009) 104:115–49. doi: 10.1016/S0065-2776(08)04004-2 20457117

[6] Harrison RA. The properdin pathway: An “alternative activation pathway” or a “critical amplification loop” for C3 and C5 activation? In: Seminars in immunopathology. (2018) 40(1):15–35. doi: 10.1007/s00281-017-0661-x 29167939

[7] Kouser L Abdul-Aziz M Nayak A Stover C Sim R Kishore U. Properdin and factor h: Opposing players on the alternative complement pathway “See-saw”. Front Immunol (2013) 4. doi: 10.3389/fimmu.2013.00093 PMC3632793 23630525

[8] Podack ER Biesecker G Muller-Eberhard HJ. Membrane attack complex of complement: generation of high-affinity phospholipid binding sites by fusion of five hydrophilic plasma proteins. Proc Natl Acad Sci U.S.A. (1979) 76:897–901. doi: 10.1073/pnas.76.2.897 284414 PMC383086

[9] Koopman JJE Van Essen MF Rennke HG De Vries APJ Van Kooten C. Deposition of the membrane attack complex in healthy and diseased human kidneys. Front Immunol (2020) 11:599974. doi: 10.3389/fimmu.2020.599974 33643288 PMC7906018

[10] Markiewski MM Lambris JD. The role of complement in inflammatory diseases from behind the scenes into the spotlight. Am J Pathol (2007) 171:715–27. doi: 10.2353/ajpath.2007.070166 PMC1959484 17640961

[11] Dunkelberger JR Song WC. Complement and its role in innate and adaptive immune responses. Cell Res (2010) 20:34–50. doi: 10.1038/cr.2009.139 20010915

[12] Vandendriessche S Cambier S Proost P Marques PE. Complement receptors and their role in leukocyte recruitment and phagocytosis. Front Cell Dev Biol (2021) 9:624025. doi: 10.3389/fcell.2021.624025 33644062 PMC7905230

[13] Daffern PJ Pfeifer PH Ember JA Hugli TE. C3a is a chemotaxin for human eosinophils but not for neutrophils. i. C3a stimulation of neutrophils is secondary to eosinophil activation. J Exp Med (1995) 181:2119–27. doi: 10.1084/jem.181.6.2119 PMC2192052 7760001

[14] Hartmann K Henz BM Krüger-Krasagakes S Köhl J Burger R Guhl S. C3a and C5a stimulate chemotaxis of human mast cells. Blood (1997) 89:2863–70. doi: 10.1182/blood.V89.8.2863 9108406

[15] Zwirner J Werfel T Wilken HC Theile E Götze O. Anaphylatoxin C3a but not C3a(desArg) is a chemotaxin for the mouse macrophage cell line J774. Eur J Immunol (1998) 28:1570–7. doi: 10.1002/(SICI)1521-4141(199805)28:05<1570::AID-IMMU1570>3.0.CO;2-6 9603462

[16] Zwirner J Götze O Begemann G Kapp A Kirchhoff K Werfel T. Evaluation of C3a receptor expression on human leucocytes by the use of novel monoclonal antibodies. Immunology (1999) 97:166–72. doi: 10.1046/j.1365-2567.1999.00764.x PMC2326815 10447728

[17] Morgan EL Ember JA Sanderson SD Scholz W Buchner R Ye RD. Anti-C5a receptor antibodies. characterization of neutralizing antibodies specific for a peptide, C5aR-(9-29), derived from the predicted amino-terminal sequence of the human C5a receptor. J Immunol (1993) 151:377–88. 8326131

[18] Sozzani S Sallusto F Luini W Zhou D Piemonti L Allavena P. Migration of dendritic cells in response to formyl peptides, C5a, and a distinct set of chemokines. J Immunol (1995) 155:3292–5. 7561021

[19] Nataf S Davoust N Ames RS Barnum SR. Human T cells express the C5a receptor and are chemoattracted to C5a. J Immunol (1999) 162:4018–23. 10201923

[20] Ottonello L Corcione A Tortolina G Airoldi I Albesiano E Favre A. rC5a directs the *in vitro* migration of human memory and naive tonsillar b lymphocytes: implications for b cell trafficking in secondary lymphoid tissues. J Immunol (1999) 162:6510–7. 10352266

[21] Li XX Lee JD Kemper C Woodruff TM. The complement receptor C5aR2: A powerful modulator of innate and adaptive immunity. J Immunol (2019) 202:3339–48. doi: 10.4049/jimmunol.1900371 31160390

[22] Oliveira LC Kretzschmar GC Dos Santos ACM Camargo CM Nisihara RM Farias TDJ. Complement receptor 1 (CR1, CD35) polymorphisms and soluble CR1: A proposed anti-inflammatory role to quench the fire of "Fogo selvagem" pemphigus foliaceus. Front Immunol (2019) 10:2585. doi: 10.3389/fimmu.2019.02585 31824479 PMC6883348

[23] Danielsson C Pascual M French L Steiger G Schifferli JA. Soluble complement receptor type 1 (CD35) is released from leukocytes by surface cleavage. Eur J Immunol (1994) 24:2725–31. doi: 10.1002/eji.1830241123 7957565

[24] Iida K Nadler L Nussenzweig V. Identification of the membrane receptor for the complement fragment C3d by means of a monoclonal antibody. J Exp Med (1983) 158:1021–33. doi: 10.1084/jem.158.4.1021 PMC2187368 6225820

[25] Reynes M Aubert JP Cohen JH Audouin J Tricottet V Diebold J. Human follicular dendritic cells express CR1, CR2, and CR3 complement receptor antigens. J Immunol (1985) 135:2687–94. 2411809

[26] Kalli KR Ahearn JM Fearon DT. Interaction of iC3b with recombinant isotypic and chimeric forms of CR2. J Immunol (1991) 147:590–4. 1830068

[27] Kovács KG Mácsik-Valent B Matkó J Bajtay Z Erdei A. Revisiting the coreceptor function of complement receptor type 2 (CR2, CD21); coengagement with the b-cell receptor inhibits the activation, proliferation, and antibody production of human b cells. Front Immunol (2021) 12:620427. doi: 10.3389/fimmu.2021.620427 33868238 PMC8047317

[28] Jensen RK Bajic G Sen M Springer TA Vorup-Jensen T Andersen GR. Complement receptor 3 forms a compact high-affinity complex with iC3b. J Immunol (2021) 206:3032–42. doi: 10.4049/jimmunol.2001208 34117107

[29] Helmy KY Katschke KJ Jr. Gorgani NN Kljavin NM Elliott JM Diehl L. CRIg: a macrophage complement receptor required for phagocytosis of circulating pathogens. Cell (2006) 124:915–27. doi: 10.1016/j.cell.2005.12.039 16530040

[30] Wiesmann C Katschke KJ Yin J Helmy KY Steffek M Fairbrother WJ. Structure of C3b in complex with CRIg gives insights into regulation of complement activation. Nature (2006) 444:217–20. doi: 10.1038/nature05263 17051150

[31] Davis AE Mejia P Lu F. Biological activities of C1 inhibitor. Mol Immunol (2008) 45:4057–63. doi: 10.1016/j.molimm.2008.06.028 PMC2626406 18674818

[32] Medof ME Kinoshita T Nussenzweig V. Inhibition of complement activation on the surface of cells after incorporation of decay-accelerating factor (DAF) into their membranes. J Exp Med (1984) 160:1558–78. doi: 10.1084/jem.160.5.1558 PMC2187498 6238120

[33] Brodbeck WG Kuttner-Kondo L Mold C Medof ME. Structure/function studies of human decay-accelerating factor. Immunology (2000) 101:104–11. doi: 10.1046/j.1365-2567.2000.00086.x PMC2327052 11012760

[34] Nicholson-Weller A Burge J Fearon DT Weller PF Austen KF. Isolation of a human erythrocyte membrane glycoprotein with decay-accelerating activity for C3 convertases of the complement system. J Immunol (1982) 129:184–9. 6211481

[35] Huang Y Qiao F Abagyan R Hazard S Tomlinson S. Defining the CD59-C9 binding interaction. J Biol Chem (2006) 281:27398–404. doi: 10.1074/jbc.M603690200 16844690

[36] Das N Biswas B Khera R. Membrane-bound complement regulatory proteins as biomarkers and potential therapeutic targets for SLE. Adv Exp Med Biol (2013) 735:55–81. doi: 10.1007/978-1-4614-4118-2_4 23402019

[37] Horwitz JK Chun NH Heeger PS. Complement and transplantation: From new mechanisms to potential biomarkers and novel treatment strategies. Clin Lab Med (2019) 39:31–43. doi: 10.1016/j.cll.2018.10.004 30709507 PMC6361534

[38] Loupy A Lefaucheur C Vernerey D Prugger C Duong Van Huyen JP Mooney N. Complement-binding anti-HLA antibodies and kidney-allograft survival. N Engl J Med (2013) 369:1215–26. doi: 10.1056/NEJMoa1302506 24066742

[39] Farrar CA Asgari E Schwaeble WJ Sacks SH. Which pathways trigger the role of complement in ischaemia/reperfusion injury? Front Immunol (2012) 3:341. doi: 10.3389/fimmu.2012.00341 23181062 PMC3500775

[40] Cravedi P Heeger PS. Complement as a multifaceted modulator of kidney transplant injury. J Clin Invest (2014) 124:2348–54. doi: 10.1172/JCI72273 PMC4038571 24892709

[41] Danobeitia JS Djamali A Fernandez LA. The role of complement in the pathogenesis of renal ischemia-reperfusion injury and fibrosis. Fibrogenesis Tissue Repair (2014) 7:16. doi: 10.1186/1755-1536-7-16 25383094 PMC4224961

[42] Howard MC Nauser CL Farrar CA Sacks SH. Complement in ischaemia-reperfusion injury and transplantation. Semin Immunopathol (2021) 43:789–97. doi: 10.1007/s00281-021-00896-3 PMC8579729 34757496

[43] Manook M Kwun J Sacks S Dorling A Mamode N Knechtle S. Innate networking: Thrombotic microangiopathy, the activation of coagulation and complement in the sensitized kidney transplant recipient. Transplant Rev (Orlando) (2018) 32:119–26. doi: 10.1016/j.trre.2018.01.001 PMC6497150 29935708

[44] Avila A Gavela E Sancho A. Thrombotic microangiopathy after kidney transplantation: An underdiagnosed and potentially reversible entity. Front Med (Lausanne) (2021) 8:642864. doi: 10.3389/fmed.2021.642864 33898482 PMC8063690

[45] Palma LMP Sridharan M Sethi S. Complement in secondary thrombotic microangiopathy. Kidney Int Rep (2021) 6:11–23. doi: 10.1016/j.ekir.2020.10.009 33102952 PMC7575444

[46] Racusen LC Colvin RB Solez K Mihatsch MJ Halloran PF Campbell PM. Antibody-mediated rejection criteria - an addition to the banff 97 classification of renl allograft rejection. Am J Transplant (2003) 3:708–14. doi: 10.1034/j.1600-6143.2003.00072.x 12780562

[47] Sellares J De Freitas DG Mengel M Reeve J Einecke G Sis B. Understanding the causes of kidney transplant failure: The dominant role of antibody-mediated rejection and nonadherence. Am J Transplant (2012) 12:388–99. doi: 10.1111/j.1600-6143.2011.03840.x 22081892

[48] Haas M Loupy A Lefaucheur C Roufosse C Glotz D Seron D. The banff 2017 kidney meeting report: Revised diagnostic criteria for chronic active T cell-mediated rejection, antibody-mediated rejection, and prospects for integrative endpoints for next-generation clinical trials. Am J Transplant (2018) 18:293–307. doi: 10.1111/ajt.14625 29243394 PMC5817248

[49] Bouquegneau A Loheac C Aubert O Bouatou Y Viglietti D Empana JP. Complement-activating donor-specific anti-HLA antibodies and solid organ transplant survival: A systematic review and meta-analysis. PloS Med (2018) 15:e1002572. doi: 10.1371/journal.pmed.1002572 29799874 PMC5969739

[50] Sicard A Ducreux S Rabeyrin M Couzi L Mcgregor B Badet L. Detection of C3d-binding donor-specific anti-HLA antibodies at diagnosis of humoral rejection predicts renal graft loss. J Am Soc Nephrol (2015) 26:457–67. doi: 10.1681/ASN.2013101144 PMC4310653 25125383

[51] Viglietti D Loupy A Vernerey D Bentlejewski C Gosset C Aubert O. Value of donor-specific anti-HLA antibody monitoring and characterization for risk stratification of kidney allograft loss. J Am Soc Nephrol (2017) 28:702–15. doi: 10.1681/ASN.2016030368 PMC5280026 27493255

[52] Viglietti D Bouatou Y Kheav VD Aubert O Suberbielle-Boissel C Glotz D. Complement-binding anti-HLA antibodies are independent predictors of response to treatment in kidney recipients with antibody-mediated rejection. Kidney Int (2018) 94:773–87. doi: 10.1016/j.kint.2018.03.015 29801667

[53] Cumpelik A Heja D Hu Y Varano G Ordikhani F Roberto MP. Dynamic regulation of b cell complement signaling is integral to germinal center responses. Nat Immunol (2021) 22:757–68. doi: 10.1038/s41590-021-00926-0 PMC8297556 34031614

[54] Serinsoz E Bock O Gwinner W Schwarz A Haller H Kreipe H. Local complement C3 expression is upregulated in humoral and cellular rejection of renal allografts. Am J Transplant (2005) 5:1490–4. doi: 10.1111/j.1600-6143.2005.00873.x 15888059

[55] Vonbrunn E Ries T Sollner S Muller-Deile J Buttner-Herold M Amann K. Multiplex gene analysis reveals T-cell and antibody-mediated rejection-specific upregulation of complement in renal transplants. Sci Rep (2021) 11:15464. doi: 10.1038/s41598-021-94954-3 34326417 PMC8322413

[56] Strainic MG Liu J Huang D An F Lalli PN Muqim N. Locally produced complement fragments C5a and C3a provide both costimulatory and survival signals to naive CD4+ T cells. Immunity (2008) 28:425–35. doi: 10.1016/j.immuni.2008.02.001 PMC2646383 18328742

[57] Lalli PN Strainic MG Yang M Lin F Medof ME Heeger PS. Locally produced C5a binds to T cell-expressed C5aR to enhance effector T-cell expansion by limiting antigen-induced apoptosis. Blood (2008) 112:1759–66. doi: 10.1182/blood-2008-04-151068 PMC2518884 18567839

[58] Pavlov V Raedler H Yuan S Leisman S Kwan WH Lalli PN. Donor deficiency of decay-accelerating factor accelerates murine T cell-mediated cardiac allograft rejection. J Immunol (2008) 181:4580–9. doi: 10.4049/jimmunol.181.7.4580 PMC2646462 18802060

[59] Strainic MG Shevach EM An F Lin F Medof ME. Absence of signaling into CD4(+) cells *via* C3aR and C5aR enables autoinductive TGF-beta1 signaling and induction of Foxp3(+) regulatory T cells. Nat Immunol (2013) 14:162–71. doi: 10.1038/ni.2499 PMC4144047 23263555

[60] Hu M Rogers NM Li J Zhang GY Wang YM Shaw K. Antigen specific regulatory T cells in kidney transplantation and other tolerance settings. Front Immunol (2021) 12:717594. doi: 10.3389/fimmu.2021.717594 34512640 PMC8428972

[61] Llaudo I Fribourg M Medof ME Conde P Ochando J Heeger PS. C5aR1 regulates migration of suppressive myeloid cells required for costimulatory blockade-induced murine allograft survival. Am J Transplant (2019) 19:633–45. doi: 10.1111/ajt.15072 PMC6375810 30106232

[62] Kilgore KS Schmid E Shanley TP Flory CM Maheswari V Tramontini NL. Sublytic concentrations of the membrane attack complex of complement induce endothelial interleukin-8 and monocyte chemoattractant protein-1 through nuclear factor-kappa b activation. Am J Pathol (1997) 150:2019–31. PMC1858311 9176395

[63] Shankland SJ Pippin JW Couser WG. Complement (C5b-9) induces glomerular epithelial cell DNA synthesis but not proliferation *in vitro* . Kidney Int (1999) 56:538–48. doi: 10.1046/j.1523-1755.1999.00560.x 10432393

[64] Brunn GJ Saadi S Platt JL. Differential regulation of endothelial cell activation by complement and interleukin 1alpha. Circ Res (2006) 98:793–800. doi: 10.1161/01.RES.0000216071.87981.16 16514066 PMC1513160

[65] Craig TJ Schneider LC Macginnitie AJ. Plasma-derived C1-INH for managing hereditary angioedema in pediatric patients: A systematic review. Pediatr Allergy Immunol (2015) 26:537–44. doi: 10.1111/pai.12425 26111105

[66] Kaabak M Babenko N Shapiro R Zokoyev A Dymova O Kim E. A prospective randomized, controlled trial of eculizumab to prevent ischemia-reperfusion injury in pediatric kidney transplantation. Pediatr Transplant (2018) 22. doi: 10.1111/petr.13129 29377474

[67] Schroppel B Akalin E Baweja M Bloom RD Florman S Goldstein M. Peritransplant eculizumab does not prevent delayed graft function in deceased donor kidney transplant recipients: Results of two randomized controlled pilot trials. Am J Transplant (2020) 20:564–72. doi: 10.1111/ajt.15580 31452319

[68] Berger M Lefaucheur C Jordan SC. Update on C1 esterase inhibitor in human solid organ transplantation. Transplantation (2019) 103:1763–75. doi: 10.1097/TP.0000000000002717 30946220

[69] Vo AA Zeevi A Choi J Cisneros K Toyoda M Kahwaji J. A phase I/II placebo-controlled trial of C1-inhibitor for prevention of antibody-mediated rejection in HLA sensitized patients. Transplantation (2015) 99:299–308. doi: 10.1097/TP.0000000000000592 25606785

[70] Montgomery RA Orandi BJ Racusen L Jackson AM Garonzik-Wang JM Shah T. Plasma-derived C1 esterase inhibitor for acute antibody-mediated rejection following kidney transplantation: Results of a randomized double-blind placebo-controlled pilot study. Am J Transplant (2016) 16:3468–78. doi: 10.1111/ajt.13871 27184779

[71] Viglietti D Gosset C Loupy A Deville L Verine J Zeevi A. C1 inhibitor in acute antibody-mediated rejection nonresponsive to conventional therapy in kidney transplant recipients: A pilot study. Am J Transplant (2016) 16:1596–603. doi: 10.1111/ajt.13663 26693703

[72] Eskandary F Jilma B Muhlbacher J Wahrmann M Regele H Kozakowski N. Anti-C1s monoclonal antibody BIVV009 in late antibody-mediated kidney allograft rejection-results from a first-in-patient phase 1 trial. Am J Transplant (2018) 18:916–26. doi: 10.1111/ajt.14528 28980446

[73] Locke JE Magro CM Singer AL Segev DL Haas M Hillel AT. The use of antibody to complement protein C5 for salvage treatment of severe antibody-mediated rejection. Am J Transplant (2009) 9:231–5. doi: 10.1111/j.1600-6143.2008.02451.x 18976298

[74] Orandi BJ Zachary AA Dagher NN Bagnasco SM Garonzik-Wang JM Van Arendonk KJ. Eculizumab and splenectomy as salvage therapy for severe antibody-mediated rejection after HLA-incompatible kidney transplantation. Transplantation (2014) 98:857–63. doi: 10.1097/TP.0000000000000298 25121475

[75] Stegall MD Diwan T Raghavaiah S Cornell LD Burns J Dean PG. Terminal complement inhibition decreases antibody-mediated rejection in sensitized renal transplant recipients. Am J Transplant (2011) 11:2405–13. doi: 10.1111/j.1600-6143.2011.03757.x 21942930

[76] Cornell LD Schinstock CA Gandhi MJ Kremers WK Stegall MD. Positive crossmatch kidney transplant recipients treated with eculizumab: outcomes beyond 1 year. Am J Transplant (2015) 15:1293–302. doi: 10.1111/ajt.13168 25731800

[77] Marks WH Mamode N Montgomery RA Stegall MD Ratner LE Cornell LD. Safety and efficacy of eculizumab in the prevention of antibody-mediated rejection in living-donor kidney transplant recipients requiring desensitization therapy: A randomized trial. Am J Transplant (2019) 19:2876–88. doi: 10.1111/ajt.15364 PMC6790671 30887675

[78] Glotz D Russ G Rostaing L Legendre C Tufveson G Chadban S. Safety and efficacy of eculizumab for the prevention of antibody-mediated rejection after deceased-donor kidney transplantation in patients with preformed donor-specific antibodies. Am J Transplant (2019) 19:2865–75. doi: 10.1111/ajt.15397 PMC9328661 31012541

[79] Pratt JR Basheer SA Sacks SH. Local synthesis of complement component C3 regulates acute renal transplant rejection. Nat Med (2002) 8:582–7. doi: 10.1038/nm0602-582 12042808

[80] Prechl J Erdei A. Immunomodulatory functions of murine CR1/2. Immunopharmacology (2000) 49:117–24. doi: 10.1016/S0162-3109(00)80297-0 10904111

[81] Malassagne B Regimbeau JM Taboit F Troalen F Chéreau C Moiré N. A new inhibitor of human complement for the prevention of xenogeneic hyperacute rejection. Xenotransplantation (2003) 10:267–77. doi: 10.1034/j.1399-3089.2003.02030.x 12694547

[82] Wang H Jiang J Liu W Kubelik D Chen G Gies D. Prevention of acute vascular rejection by a functionally blocking anti-C5 monoclonal antibody combined with cyclosporine. Transplantation (2005) 79:1121–7. doi: 10.1097/01.TP.0000161218.58276.9A 15880054

[83] Wang H Arp J Liu W Faas SJ Jiang J Gies DR. Inhibition of terminal complement components in presensitized transplant recipients prevents antibody-mediated rejection leading to long-term graft survival and accommodation. J Immunol (2007) 179:4451–63. doi: 10.4049/jimmunol.179.7.4451 17878341

[84] Raedler H Vieyra MB Leisman S Lakhani P Kwan W Yang M. Anti-complement component C5 mAb synergizes with CTLA4Ig to inhibit alloreactive T cells and prolong cardiac allograft survival in mice. Am J Transplant (2011) 11:1397–406. doi: 10.1111/j.1600-6143.2011.03561.x PMC3128644 21668627

[85] Lin T Zhou W Farrar CA Hargreaves RE Sheerin NS Sacks SH. Deficiency of C4 from donor or recipient mouse fails to prevent renal allograft rejection. Am J Pathol (2006) 168:1241–8. doi: 10.2353/ajpath.2006.050360 PMC1606553 16565498

[86] Xie CB Jane-Wit D Pober JS. Complement membrane attack complex: New roles, mechanisms of action, and therapeutic targets. Am J Pathol (2020) 190:1138–50. doi: 10.1016/j.ajpath.2020.02.006 PMC7280757 32194049

[87] Qian Z Hu W Liu J Sanfilippo F Hruban RH Baldwin WM. Accelerated graft arteriosclerosis in cardiac transplants: Complement activation promotes progression of lesions from medium to large arteries. Transplantation (2001) 72:900–6. doi: 10.1097/00007890-200109150-00027 11571457

[88] Suhr BD Black SM Guzman-Paz M Matas AJ Dalmasso AP. Inhibition of the membrane attack complex of complement for induction of accommodation in the hamster-to-rat heart transplant model. Xenotransplantation (2007) 14:572–9. doi: 10.1111/j.1399-3089.2007.00422.x 17991145

[89] Fu C Shi L Huang X Feng H Tan X Chen S. A novel metalloprotease with anti-complement and anti-coagulant activity, significantly delays discordant cardiac xenograft rejection. Xenotransplantation (2020) 27:e12616. doi: 10.1111/xen.12616 32529740

[90] Lin K Zhang L Kong M Yang M Chen Y Poptic E. Development of an anti-human complement C6 monoclonal antibody that inhibits the assembly of membrane attack complexes. Blood Adv (2020) 4:2049–57. doi: 10.1182/bloodadvances.2020001690 PMC7218433 32396613

[91] Merle NS Noe R Halbwachs-Mecarelli L Fremeaux-Bacchi V Roumenina LT. Complement system part II: Role in immunity. Front Immunol (2015) 6:257. doi: 10.3389/fimmu.2015.00257 26074922 PMC4443744

[92] Mathern DR. Absence of recipient C3aR1 signaling limits expansion and differentiation of alloreactive CD8(+) T cell immunity and prolongs murine cardiac allograft survival. Am J Transplant (2019) 19:1628–40. doi: 10.1111/ajt.15222 PMC6538425 30565852

[93] Li Q Peng Q Xing G Li K Wang N Farrar CA. Deficiency of C5aR prolongs renal allograft survival. J Am Soc Nephrol (2010) 21:1344–53. doi: 10.1681/ASN.2009090977 PMC2938594 20651167

[94] Gueler F Rong S Gwinner W Mengel M Brocker V Schon S. Complement 5a receptor inhibition improves renal allograft survival. J Am Soc Nephrol (2008) 19:2302–12. doi: 10.1681/ASN.2007111267 PMC2588101 18753257

[95] Schmitz R Fitch ZW Schroder PM Choi AY Manook M Yoon J. C3 complement inhibition prevents antibody-mediated rejection and prolongs renal allograft survival in sensitized non-human primates. Nat Commun (2021) 12:5456. doi: 10.1038/s41467-021-25745-7 34526511 PMC8443599

[96] Chen Song S Zhong S Xiang Y Li JH Guo H Wang WY. Complement inhibition enables renal allograft accommodation and long-term engraftment in presensitized nonhuman primates. Am J Transplant (2011) 11:2057–66. doi: 10.1111/j.1600-6143.2011.03646.x 21831160

[97] Chen G Sun QY Wang XM Shen SQ Guo H Wang H. Improved suppression of circulating complement does not block acute vascular rejection of pig-to-rhesus monkey cardiac transplants. Xenotransplantation (2004) 11:123–32. doi: 10.1111/j.1399-3089.2004.00048.x 14962274

[98] Tillou X Poirier N Le Bas-Bernardet S Hervouet J Minault D Renaudin K. Recombinant human C1-inhibitor prevents acute antibody-mediated rejection in alloimmunized baboons. Kidney Int (2010) 78:152–9. doi: 10.1038/ki.2010.75 20336054

[99] Eerhart MJ Reyes JA Blanton CL Danobeitia JS Chlebeck PJ Zitur LJ. Complement blockade in recipients prevents delayed graft function and delays antibody-mediated rejection in a nonhuman primate model of kidney transplantation. Transplantation (2022) 106:60–71. doi: 10.1097/TP.0000000000003754 34905763 PMC8674492

[100] Miyagawa S Takeishi S Yamamoto A Ikeda K Matsunari H Yamada M. Survey of glycoantigens in cells from alpha1-3galactosyltransferase knockout pig using a lectin microarray. Xenotransplantation (2010) 17:61–70. doi: 10.1111/j.1399-3089.2009.00565.x 20149189

[101] Oriol R Ye Y Koren E Cooper DK. Carbohydrate antigens of pig tissues reacting with human natural antibodies as potential targets for hyperacute vascular rejection in pig-to-man organ xenotransplantation. Transplantation (1993) 56:1433–42. doi: 10.1097/00007890-199312000-00031 8279016

[102] Chen G Qian H Starzl T Sun H Garcia B Wang X. Acute rejection is associated with antibodies to non-gal antigens in baboons using gal-knockout pig kidneys. Nat Med (2005) 11:1295–8. doi: 10.1038/nm1330 PMC3018862 16311604

[103] Byrne GW Stalboerger PG Du Z Davis TR Mcgregor CG. Identification of new carbohydrate and membrane protein antigens in cardiac xenotransplantation. Transplantation (2011) 91:287–92. doi: 10.1097/TP.0b013e318203c27d PMC10022691 21119562

[104] Kuwaki K Tseng YL Dor FJ Shimizu A Houser SL Sanderson TM. Heart transplantation in baboons using alpha1,3-galactosyltransferase gene-knockout pigs as donors: initial experience. Nat Med (2005) 11:29–31. doi: 10.1038/nm1171 15619628

[105] Tseng YL Kuwaki K Dor FJ Shimizu A Houser S Hisashi Y. alpha1,3-galactosyltransferase gene-knockout pig heart transplantation in baboons with survival approaching 6 months. Transplantation (2005) 80:1493–500. doi: 10.1097/01.tp.0000181397.41143.fa 16340796

[106] Lutz AJ Li P Estrada JL Sidner RA Chihara RK Downey SM. Double knockout pigs deficient in n-glycolylneuraminic acid and galactose alpha-1,3-galactose reduce the humoral barrier to xenotransplantation. Xenotransplantation (2013) 20:27–35. doi: 10.1111/xen.12019 23384142

[107] Li Q Shaikh S Iwase H Long C Lee W Zhang Z. Carbohydrate antigen expression and anti-pig antibodies in new world capuchin monkeys: Relevance to studies of xenotransplantation. Xenotransplantation (2019) 26:e12498. doi: 10.1111/xen.12498 30770572 PMC6591082

[108] Byrne GW Du Z Stalboerger P Kogelberg H Mcgregor CG. Cloning and expression of porcine beta1,4 n-acetylgalactosaminyl transferase encoding a new xenoreactive antigen. Xenotransplantation (2014) 21:543–54. doi: 10.1111/xen.12124 PMC4262693 25176027

[109] Estrada JL Martens G Li P Adams A Newell KA Ford ML. Evaluation of human and non-human primate antibody binding to pig cells lacking GGTA1/CMAH/beta4GalNT2 genes. Xenotransplantation (2015) 22:194–202. doi: 10.1111/xen.12161 25728481 PMC4464961

[110] Ladowski JM Reyes LM Martens GR Butler JR Wang ZY Eckhoff DE. Swine leukocyte antigen class II is a xenoantigen. Transplantation (2018) 102:249–54. doi: 10.1097/TP.0000000000001924 PMC5978411 28846555

[111] Martens GR Ladowski JM Estrada J Wang ZY Reyes LM Easlick J. HLA class I-sensitized renal transplant patients have antibody binding to SLA class I epitopes. Transplantation (2019) 103:1620–9. doi: 10.1097/TP.0000000000002739 30951017

[112] Adams AB Lovasik BP Faber DA Burlak C Breeden C Estrada JL. Anti-C5 antibody tesidolumab reduces early antibody-mediated rejection and prolongs survival in renal xenotransplantation. Ann Surg (2021) 274:473–80. doi: 10.1097/SLA.0000000000004996 PMC8915445 34238812

[113] Cozzi E Langford GA Richards A Elsome K Lancaster R Chen P. Expression of human decay accelerating factor in transgenic pigs. Transplant Proc (1994) 26:1402–3. 7518135

[114] Cozzi E Vial C Ostlie D Farah B Chavez G Smith KG. Maintenance triple immunosuppression with cyclosporin a, mycophenolate sodium and steroids allows prolonged survival of primate recipients of hDAF porcine renal xenografts. Xenotransplantation (2003) 10:300–10. doi: 10.1034/j.1399-3089.2003.02014.x 12795679

[115] Yamada K Yazawa K Shimizu A Iwanaga T Hisashi Y Nuhn M. Marked prolongation of porcine renal xenograft survival in baboons through the use of alpha1,3-galactosyltransferase gene-knockout donors and the cotransplantation of vascularized thymic tissue. Nat Med (2005) 11:32–4. doi: 10.1038/nm1172 15619627

[116] Diamond LE Mccurry KR Martin MJ Mcclellan SB Oldham ER Platt JL. Characterization of transgenic pigs expressing functionally active human CD59 on cardiac endothelium. Transplantation (1996) 61:1241–9. doi: 10.1097/00007890-199604270-00021 8610425

[117] Diamond LE Quinn CM Martin MJ Lawson J Platt JL Logan JS. A human CD46 transgenic pig model system for the study of discordant xenotransplantation. Transplantation (2001) 71:132–42. doi: 10.1097/00007890-200101150-00021 11211178

[118] Mcgregor CG Ricci D Miyagi N Stalboerger PG Du Z Oehler EA. Human CD55 expression blocks hyperacute rejection and restricts complement activation in gal knockout cardiac xenografts. Transplantation (2012) 93:686–92. doi: 10.1097/TP.0b013e3182472850 PMC3314133 22391577

[119] Azimzadeh AM Kelishadi SS Ezzelarab MB Singh AK Stoddard T Iwase H. Early graft failure of GalTKO pig organs in baboons is reduced by expression of a human complement pathway-regulatory protein. Xenotransplantation (2015) 22:310–6. doi: 10.1111/xen.12176 PMC5172381 26174749

[120] Mohiuddin MM Singh AK Corcoran PC Thomas Iii ML Clark T Lewis BG. Chimeric 2C10R4 anti-CD40 antibody therapy is critical for long-term survival of GTKO.hCD46.hTBM pig-to-primate cardiac xenograft. Nat Commun (2016) 7:11138. doi: 10.1038/ncomms11138 27045379 PMC4822024

[121] Adams AB Kim SC Martens GR Ladowski JM Estrada JL Reyes LM. Xenoantigen deletion and chemical immunosuppression can prolong renal xenograft survival. Ann Surg (2018) 268:564–73. doi: 10.1097/SLA.0000000000002977 PMC6382078 30048323

[122] Vangerow B Hecker JM Lorenz R Loss M Przemeck M Appiah R. C1-inhibitor for treatment of acute vascular xenograft rejection in cynomolgus recipients of h-DAF transgenic porcine kidneys. Xenotransplantation (2001) 8:266–72. doi: 10.1034/j.1399-3089.2001.00130.x 11737852

[123] Zelek WM Morgan BP. Monoclonal antibodies capable of inhibiting complement downstream of C5 in multiple species. Front Immunol (2020) 11:612402. doi: 10.3389/fimmu.2020.612402 33424866 PMC7793867

